# Radiation Safety and External Radiation Exposure Rate of Patients Receiving I-131 Therapy for Hyperthyroidism and Remnant Ablation as Outpatient: An Institutional Experience

**DOI:** 10.1055/s-0043-1771285

**Published:** 2023-09-06

**Authors:** Nisha Bhatia, Vandana K. Dhingra, Pulkit Mittal, Sunil Saini

**Affiliations:** 1 Department of Nuclear Medicine, Cancer Research Institute, Swami Rama Himalayan University (SRHU), Dehradun, Uttarakhand, India; 2 Department of Nuclear Medicine, All India Institute of Medical Sciences (AIIMS), Rishikesh, Uttarakhand, India; 3 Department of Surgical Oncology, Cancer Research Institute, Swami Rama Himalayan University (SRHU) Dehradun, Uttarakhand, India

**Keywords:** radioiodine therapy, thyrotoxicosis, ablation, outpatient, radiation exposure

## Abstract

**Objective** Our objective was to study the radiation exposure rate as function of time in the administration of radioiodine iodine-131 (I-131) for the treatment of thyrotoxicosis or Graves' disease and remnant ablation on an outpatient basis at the Department of Nuclear Medicine, and also, to study the impact of revised discharge criteria for radioiodine therapy enforced by the Atomic Energy Regulatory Board (AERB) of India.

**Materials and Methods** This study included patients who underwent low-dose radioiodine therapy using I-131. Patients were classified into two different groups, that is, group A and group B. Group A included patients receiving low dose I-131 for the treatment of thyrotoxicosis, whereas group B included patients receiving I-131 therapy for the ablation of residual thyroid tissue after total thyroidectomy. The radiation exposure rate was measured using a radiation detector in milli roentgen per hour (mR/h) at 5 cm distance of stomach and neck levels and with the patient standing at the distance of 1 m after oral administration of I-131 at 0, 1, and 2 hours.

**Results** A total of 134 (17 males and 117 females) patients were included in the study. Group A comprised 102 (14 male and 88 females) patients and group B of 32 (3 males and 29 females) patients. At the neck level, the average exposure rate in group A versus group B after 0, 1, and 2 hours was observed to be 6.9 versus 22.27 mR/h, 33.67 versus 43.39 mR/h, and 41.75 versus 48.90 mR/h, respectively. At the stomach level, the exposure rate was 23.65 versus 71.32 mR/h, 13.27 versus 48.45 mR/h, and 9.91 versus 39.43 mR/h after 0, 1, and 2 hours, respectively. At a distance of 1 m, the exposure rate was 1.31 versus 2.99 mR/h, 1.05 versus 2.58 mR/h, and 0.92 versus 2.21 mR/h, respectively.

**Conclusion** Exposure rate measured for patients treated with up to 1,110 MBq (30 mCi) of I-131 was under permissible limits as per revised discharged limits, that is, 50 µSv/h (5 mR/h) prescribed by AERB, India. The patients undergoing radioiodine therapy I-131 (up to 1,110 MBq/30 mCi) can be discharged safely 2 hours postadministration following good work practice along with providing proper radiation safety instructions to patients.

## Introduction

Since 1940s, radioiodine therapy (I-131) has been the most widely used method for the treatment of various benign and malignant thyroid disorders like hyperthyroidism and thyroid carcinoma. 1 Although the radioiodine therapy with I-131 is safe and has fewer chances of side effects, there is a major risk of external radiation hazard due to its gamma emission. 2 Because of the strong penetration power and relatively high energy of gamma radiation emitted from I-131, radiation safety becomes a major concern. Because the high energy gamma radiation can penetrate through the patient's body and can cause external radiation hazards to the persons around the patient especially to the children and pregnant females as they are more sensitive to radiation. 3 4 In radioiodine therapy, proper radiation safety practices and precautions are mandatory to minimize unnecessary radiation exposure to the family of the patient, occupational workers treating the patient, and general public. 5

Iodine-131 undergoes beta minus decay with the emission of gamma radiation of 364 keV along with the emission of high-energy beta particles, a maximum energy of 0.61 MeV, and an average energy of 0.192 MeV. Because of its long physical half-life of 8.1 days and trapping as well as organification capacity by thyroid, it is the ideal physiological radioisotope of choice for the treatment. 1 Radioiodine therapy is indicated for patients suffering from hyperthyroidism mainly due to Grave's disease, nontoxic multinodular goiter, and thyroid carcinoma. Severe uncontrolled thyrotoxicosis and in female patients pregnancy and breastfeeding are common contraindications for radioiodine therapy. 1 Therapeutic doses of I-131 commonly range from 185 MBq (5 mCi) to 11,100 MBq (300 mCi). Lower radioactivity is usually recommended to treat Grave's or toxic Goiter. Higher doses are used to ablate the remnant thyroid tissue and treat metastatic disease in patients with differentiated thyroid carcinoma. Higher doses require hospitalization of patients in specially designed isolation wards for a period until the radiation exposure rate of the patient comes under permissible discharge limits prescribed by the competent authority. These criteria for the discharge limit of the patients vary from country to country. As per the International Atomic Energy Agency (IAEA), patients undergoing radioiodine therapy could be discharged if I-131 activity is given up to 1,110 MBq (30 mCi). Earlier, according to the competent authority of India, Atomic Energy Regulatory Board (AERB), radioiodine patients could be discharged if I-131 activity given was up to 555 MBq (15 mCi) and the radiation level at the distance of 1 m from the patient did not exceed 20–30 µSv/h (2.5 mR/h) at the time of discharge. 6 In 2011, the competent authority of India revised its criteria for radioiodine patient's discharge, allowing the discharge of patients for higher I-131 activity up to 1,110 MBq (30 mCi) and the radiation level at the distance of 1 m from the patient should not exceed 50 µSv/h (5 mR/h). 7

The use of a radiation monitor is the most accepted method for the measurement of external radiation exposure of patients. 8 The aim of this study is to determine the radiation exposure rate as the function of time in outpatient administration of radioiodine I-131 in the treatment of thyrotoxicosis and remnant ablation using a radiation monitor in the region of the Himalayan range of Uttarakhand and to study the impact of revised discharge criteria for radioiodine therapy enforced by regulatory authority AERB India.

## Material and Methods

### Patients

The study was performed at the Nuclear Medicine Department, Cancer Research Institute, Swami Rama Himalayan University (SRHU) Dehradun, Uttarakhand. The patients who received low-dose radioiodine (I-131) therapy were referred from Himalayan Hospital, Swami Rama Himalayan University. Written informed consent was taken from all patients undergoing radioiodine therapy following the detailed verbal and written explanation of the whole procedure and possible associated risks involved. This study population was categorized into two groups: (group A) patients with thyrotoxicosis disease and (group B) patients for the ablation of residual thyroid tissue after total thyroidectomy.

### Patient Preparation

Antithyroid drugs like neomercazole, carbimazole were stopped 1 week before the therapy for patients suffering from thyrotoxicosis. For remnant ablation, therapy was given after 1 month of thyroidectomy. In female patients of the reproductive age group, pregnancy was ruled out and patients were counseled to avoid pregnancy for 6 to 12 months after the therapy. Pregnant females and lactating mothers were excluded from the study. Patients were instructed to take lemon in any form (juice, candy, Vitamin C tablets, etc.) to minimize the radiation dose to salivary glands. In patients, increased fluids/water intake was encouraged and frequent voiding was suggested after the therapy to minimize the radiation dose to gonads, kidneys, and urinary bladder. Patients were not allowed to eat 2 hours before and after administration of I-131 dose to minimize the radiation dose to the stomach and reduce the chances of developing nausea. Patients were also instructed to sleep alone and to avoid close contact with pregnant females and children for a period of time specified in accordance to the radioactivity dose given to the patient.

### Oral Administration of Radioiodine

The radioiodine (I-131), sodium iodide solution supplied by BRIT Mumbai, India. I-131 doses were measured in well-calibrated dose calibrator (CAPINTEC- CRC 25 R) and properly tagged with the details like activity, time, date, and patient's name. Before giving the therapy, identify the patient to avoid the misadministration. The measured and tagged I-131 dose was orally administered to the patient inside the well-ventilated fume hood. All work surfaces were properly covered with absorbent sheets backed with plastic sheets. All safety measures were taken during the handling and administration of radioiodine. In group A, the administered activity of I-131 was 207.2 ± 55.5 MBq (5.6 ± 1.5 mCi) with range 96.2 to 543.9 MBq (2.6–14.7 mCi), whereas in group B the administered activity was 725.2 ± 188.7 MBq (19.6 ± 5.1 mCi) with range 447.7 to 1091.5 MBq (12.1–29.5 mCi).

### Exposure Rate Measurement

After oral administration of radioiodine, the radiation exposure rate of the patients was measured using a radiation survey meter (Victoreen, United States, 451B-RYR) based on the ionization chamber. This is a handheld-type radiation detector with digital reading facility. The measuring range of detector was 0.1 μSv/h to 100 Sv/h. The radiation exposure rate of patient was measured in mR/h at the distance of 5 cm distance from stomach and neck levels, and at the distance of 1 m with patient standing after oral administration of I-131 at 0, 1, and 2 hours. Patients were kept under observation up to 2 hours of administration of I-131 in postadministration patient's waiting area and then allowed to go home.

## Results

A total of 134 patients (117 females and 17 males) were included in the study. The mean age of the patients was 41 years with range 10 to 75 years. The group A comprised 102 patients receiving low-dose radioiodine (I-131) therapy for patients with thyrotoxicosis, whereas group B included 32 patients receiving radioiodine therapy for the ablation of residual thyroid tissue after total thyroidectomy. The I-131 dose given to Group B was significantly higher compared to the dose given to Group A.

Group A includes 102 patients (88 females and 14 males) who underwent radioiodine therapy for thyrotoxicosis. All readings taken for the exposure rate for every patient included in the study were normalized with respect to the radioiodine activity administered to the patient to get a clear picture of radioactivity distribution pattern. Table 1 shows the normalized average exposure rate at neck, stomach, and distance of 1 m at different time intervals for patients who underwent radioiodine therapy for thyrotoxicosis (group A).

**Table 1:** Normalized average exposure rate for group A patients at neck, stomach, and distance of 1 m from patients for different time intervals where T′0 is time of I-131 administration, T′1 is 1 hour after I-131 administration, T′2 is 2 hours after I-131 administration

	T′0 (mR/h/mCi)	T′1 (mR/h/mCi)	T′2 (mR/h/mCi)
Neck	1.2	6.07	7.57
Stomach	4.2	2.43	1.8
1 m distance from patient	0.23	0.19	0.17

**Fig. 1:**
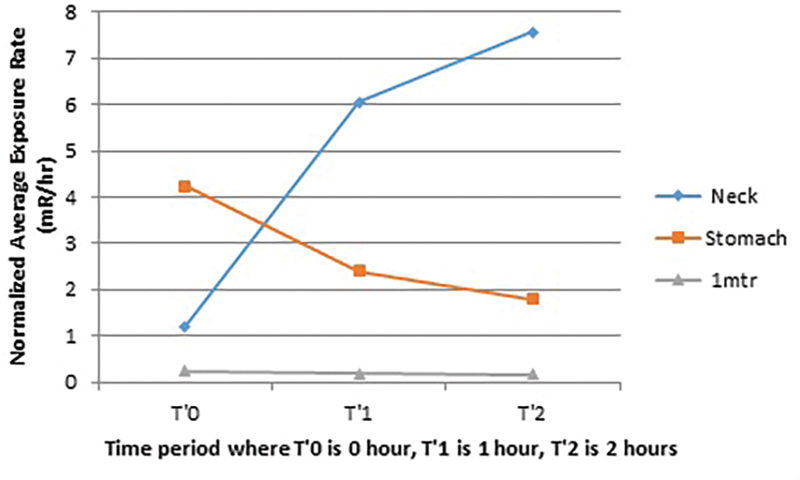
Pattern of average activity or average exposure rate at level of neck, stomach, and at the distance of 1 m from patient at the time interval of T′0, T′1, and T′2 in Group A, where T′0 time of I-131 administration, T′1 1 hour after I-131 administration, T′2 2 hours after I-131 administration.

However, Fig. 1 summarizes the pattern of average activity or the average exposure rate at the level of neck, stomach, and at 1 m distance from patient at the time interval of T′0, T′1, T′2 in group A.

**Fig. 2:**
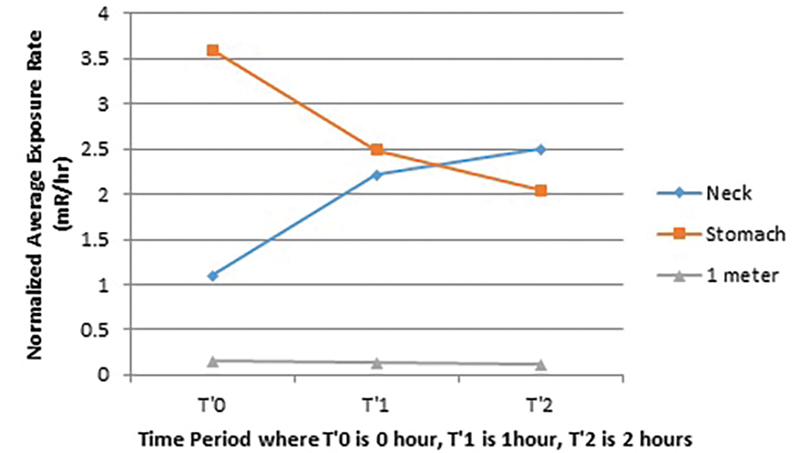
Pattern of average activity or average exposure rate at the level of neck, stomach, and at distance of 1 m from the patient at the time interval of T′0, T′1, T′2 in Group B, where T′0 time of I-131 administration, T′1 1 hour after I-131 administration, T′2 2 hours after I-131 administration.

Group B includes 32 patients (29 females and 3 males) underwent radioiodine therapy for the ablation of residual thyroid tissue after total thyroidectomy. Table 2 shows the normalized average exposure rate at neck, stomach, and distance of 1 m from patients at different time intervals of radioiodine therapy given. However, Fig. 2 summarizes the pattern of average activity or the average exposure rate at the level of neck, stomach, and at the distance of 1m from patient at the time interval of T′0, T′1, and T′2 in group B.

**Table 2:** Normalized average exposure rate for group B patients at neck, stomach and distance of 1 m from patients for different time intervals where T′0 is time of I-131 administration, T′1 is 1 hour after I-131 administration, T′2 is 2 hours after I-131 administration

	T′0 (mR/h/mCi)	T′1 (mR/h/mCi)	T′2 (mR/h/mCi)
Neck	1.1	2.21	2.5
Stomach	3.5	2.5	2.04
1 m distance from patient	0.16	0.13	0.11

This was observed that the normalized average exposure rate was lower for group B as compare to group A.

## Discussion

High-energy and penetration power of gamma emission of I-131 makes it a major source of external exposure and the volatile nature of I-131 leads to the chances of internal exposure too, by inhalation of radioactive vapors. Due to all these factors, radiation protection becomes a very sensitive issue in radioiodine therapy to avoid unnecessary exposure to occupational workers, patients, as well as general public. Guidelines of safe handling and radiation safety aspects are well outlined by IAEA reports and implemented by AERB in India. 9 10 According to the guidelines, after I-131 therapy radiation exposure should be measured in all patients before their discharge from hospital to assure that the external exposure rate from the patient underwent therapy is under permissible limits prescribed by competitive authority of AERB, India and safe for patient's family and general public.

The administered radioiodine is absorbed in the functioning thyroid tissue and gets washed out through bowels and kidneys, resulting in whole body radiation burden. The present study was designed to study the pattern of radiation exposure in patients belonging to the Himalayan region of Uttarakhand because this Himalayan region is an iodine-deficient region where a small amount of radioiodine dose can attribute to high-radiation exposure. However, the present study depicts that patients undergoing radioiodine therapy with radioactivity dose up to 1,110 MBq (30 mCi) can be safely managed in the outpatient clinic.

In group A, the initial exposure rate measured after the oral administration of I-131 at the surface of stomach was higher as compare to the exposure rate at the neck level and distance of 1 m from patient. At the time interval of 1 and 2 hours of I-131 administration, the exposure rate at the neck level was increasing and at surface of stomach the exposure rate was decreasing. This shows that iodine was slowly taken up by the thyroid gland. Similarly, the exposure rate at the distance of 1 m from patient was also decreasing, which suggests that patient can be safely discharged after 2 hours of I-131 administration as the exposure rate from patient becomes very minimal.

The same pattern of activity and exposure distribution was observed in group B as was observed in group A. But in patients treated for ablations of residual thyroid tissue after thyroidectomy in group B, radioiodine activity gets cleared from bowels at the faster rate as compared to the patients treated for thyrotoxicosis resulting into lesser radiation burden. Ravichandran et al quoted that after thyroidectomy, because of the surgical excision of the thyroid gland, radioiodine (I-131) gets washed out from the patient's body at a faster rate. 8 Hänscheid et al and Sisson et al also stated that the lack of thyroid gland and higher renal clearance in thyroid cancer patient causes faster excretion of activity and reduces the exposure rate more rapidly during radioiodine ablation. 11 12

Immediately after the oral administration of I-131, the normalized average exposure rate at the distance of 1 m distance was 0.23 and 0.16 mR/h, respectively, for thyrotoxicosis and ca thyroid patient. Exposure rate at the distance of 1 m after administering the maximum of 1,091.5 MBq (29.5 mCi) of I-131 was observed to be 4.8 mR/h which lowered to 3 mR/h after 2 hours of I-131 administration. Maximum exposure rate observed at the distance of 1 m of patient was 5.1 mR/h which lowered to 3.6 mR/h at 2 hours of I-131 administration, this patient was administered with 917.6 MBq (24.8 mCi) of I-131. These observations also emphasize practicing the 2 hours observation period posttherapy to ensure that the exposure rate is under permissible limits of discharge criteria. Also, suppose any kind of medical emergency happens post-radio-iodine dose administration. Radiation professionals can better handle it inside the hospital premises, further lowering the risk of unnecessary radiation burden to the patient's family and the general public. Results of present study showed that exposure rates measured for all patients were under permissible limits as per revised discharged limits prescribed by AERB. So the revised permissible limits prescribed by AERB India, for discharge criteria after radioiodine therapy can be implemented successfully.

## Conclusion

As the exposure rate measured for patients treated with up to 1,110 MBq (30 mCi) of I-131 was under permissible limits as per revised discharged limits prescribed by AERB, based upon our results, it is suggested that with good work practice and thorough radiation safety instructions to patients and their attendants, thyrotoxicosis patients and patients for remnant ablation treating with radioactivity dose up to (1,110 MBq) 30 mCi of I-131 can be discharged safely after 2 hours of administration of I-131 as outpatients. This practice will reduce the need for additional isolation wards and cost of treatment for hospital as well as for the patients too.
